# The Role of Pain Catastrophizing, Emotional Intelligence, and Pain Intensity in the Quality of Life of Cancer Patients with Chronic Pain

**DOI:** 10.1007/s10880-022-09921-5

**Published:** 2022-11-07

**Authors:** Fotios Anagnostopoulos, Aristi Paraponiari, Konstantinos Kafetsios

**Affiliations:** 1 Department of Psychology, Panteion University, 136, Syngrou Avenue, 176 71 Athens, Greece; 2 School of Film, Aristotle University of Thessaloniki, Thessaloniki, Greece

**Keywords:** Pain catastrophizing, Emotional intelligence, Quality of life, Chronic pain, Cancer patients

## Abstract

Pain catastrophizing (PC) is a negative cognitive distortion to actual or anticipated pain. This study aims to investigate the relationship between pain catastrophizing, emotional intelligence, pain intensity, and quality of life (QoL) in cancer patients with chronic pain. Eighty-nine outpatients with chronic pain attending pain clinics and palliative care units were recruited. Participants were men (42.7%) and women (57.3%) with an average age of 56.44 years (*SD* = 14.82). Self-report psychological measures were completed, including a measure of emotional intelligence, a standard measure of PC, a scale assessing pain intensity, and a scale measuring QoL. The PC scale was found to assess three correlated yet different dimensions of pain catastrophizing (helplessness, magnification, and rumination). Moreover, as expected, patients with PC scale scores ≥ 30 had lower scores in functional QoL dimensions and higher scores in the fatigue, pain, and insomnia symptom dimensions. Regression analyses demonstrated that PC (*B* = − 0.391, *p* = 0.004), pain intensity (*B* = − 1.133, *p* < 0.001), and education (*B* = 2.915, *p* = 0.017) remained the only significant variables related to QoL, when controlling for demographic and clinical confounders. Regarding mediating effects, PC and pain intensity were jointly found to be significant mediators in the relationship between emotional intelligence and QoL. Results are discussed in the context of the clinical implications regarding interventions designed to improve cancer patients’ quality of life and offer new insight, understanding, and evaluation targets in the field of pain management.

According to the International Association for the Study of Pain (IASP), pain is defined as an unpleasant sensory and emotional experience associated with, or resembling that associated with, actual or potential tissue damage (Raja et al., 2020). This definition acknowledges that emotional states and cognitive-perceptual processes may modulate the intensity and duration of pain as well as the whole pain experience (Lumley et al., 2011). Pain is one of the most invalidating, highly debilitating, and feared consequences of cancer (Caraceni & Shkodra, 2019) and can be caused by cancer itself (e.g., a tumor pressing on nerves), cancer treatment and treatment side effects (e.g., peripheral neuropathy, mouth sores, radiation mucositis), or other procedures and tests (Burton et al., 2007; Levy et al., 2008). Research suggests that the prevalence rate of pain in adult patients with cancer is 39.3% after curative treatment, rising to 55% for patients undergoing anti-cancer treatment, and increasing to 66.4% in patients with advanced, metastatic, or terminal disease (van den Beuken- van Everdingen, Hochstenbach, Joosten, Tjan-Heijnen, & Janssen, 2016). Nearly 10% of cancer survivors in the United States are experiencing pain related to cancer or cancer treatment that may have persisted for years after their initial diagnosis. Approximately, 20% of those experiencing pain report that pain has been inadequately controlled (Gallaway et al., 2020)**.**

Apart from acute pain, between 33 and 40% of cancer survivors suffer from chronic pain (Wilkinson & Branco, 2020), defined as persistent or recurrent pain lasting for longer than 3 months. In a recent survey involving civilian, non-institutionalized U.S. population, data were collected on chronic pain (i.e., pain on most days or every day in the past 6 months) and high-impact chronic pain (HICP; defined as chronic pain limiting life or work activities on most days or every day in the past 6 months). Among the identified cancer survivors in this survey, it was found that 34.6% reported having chronic pain and 16.1% having HICP, representing approximately 5.39 million and 2.51 million cancer survivors, respectively, in the U.S. population (Jiang et al., 2019). These prevalence figures for chronic pain and HICP among cancer survivors were almost double that in the general U.S. population (Gallaway et al., 2020).

Despite the availability of effective pharmacological and non-pharmacological therapies and evidence-based pain management guidelines, cancer pain continues to be a challenging symptom associated with inadequate pain management and the resulting decrease in quality of life (QoL) and functionality (Fisch et al., 2012; Goncalves, Veiga, & Araujo, 2021; Green et al., 2011). Cancer-related pain appears to interfere with multiple aspects of QoL of cancer patients, including daily living activities, work, mood, social function, and sleep quality (te Boveldt et al., 2013). Research has shown that pain associated with cancer is described as distressing, an intolerable aspect of cancer, creating difficulties in performing normal daily activities (Breivik et al., 2009; Vallerand et al., 2007). Thus, pain in cancer patients and reduced quality of life due to pain are matters of concern and motivated the present study. More importantly, the vast majority of relevant studies have focused on a small number of psychological variables associated with QoL in cancer patients with pain. Among these variables, anxiety, depression, stigma, hope, religiosity, and spirituality have been studied more frequently (e.g., Malak et al., 2018). Thus, there is a need to broaden this set of psychological variables and include some recently proposed factors (Leysen et al., 2021). The present study aimed to investigate the relationships between emotional intelligence (EI), pain catastrophizing (PC), pain intensity, and quality of life in cancer patients with chronic pain.

In recent years, a growing body of research has confirmed the critical role of psychological factors in the experience of pain (Day et al., 2020). Among these factors, pain catastrophizing has emerged as one of the most robust psychological predictors of pain, shown to contribute to higher levels of disability and increased pain medication use. PC is a particular negative cognitive response style related to actual or anticipated painful experiences. It is characterized by a tendency to ruminate on aspects of the painful experiences, exaggerate the threat value of the pain sensation, and adopt a helpless orientation toward pain (Bishop & Warr, 2003; Petrini & Arendt-Nielsen, 2020; Slepian et al., 2020; Sullivan et al., 1995). PC is one of the key elements of the fear-avoidance model (FAM), which has been suggested as an explanatory model for the trajectory leading to chronic pain and disability (Leeuw et al., 2007; Vlaeyen & Linton, 2012; Vlaeyen et al., 2016). According to the FAM, acute pain, possibly caused by an injury, may be appraised and interpreted as highly threatening and as a catastrophe (pain catastrophizing), while priority is given to pain control. Then, pain-related fear evolves, which leads to avoidance behaviors and increased attention toward bodily sensations and the source of the threat (hypervigilance). Consequently, daily activities, expected to produce or worsen pain, are avoided and are not accomplished anymore, leading to activity restrictions, functional incapacity, disuse, distress, depression, and negative affect. As a result, the individual becomes trapped in a vicious circle of persistent and increasing fear and dysfunctional avoidance. This pattern leads, in turn, to the experience of prolonged and chronic pain and pain-related disability, that conjointly decrease health-related quality of life and exacerbate the intensity of the pain experience (Börsbo et al., 2008; Parr et al., 2012; Poulin et al., 2016a, 2016b; Wong et al., 2014).

Gellatly and Beck (2016) have emphasized that catastrophic thinking may play a critical role across psychopathological conditions, where a precipitating event activates catastrophic beliefs. Individuals magnify the perceived threat, exaggerate the potential negative consequences of it, and imagine the worst possible outcome. Such individuals interpret neutral situations in negative ways (interpretive bias) and pay excessive attention to potentially threatening information (attentional bias). They are not able to reappraise dysfunctional cognitions (attentional fixation), while somatic symptoms (such as chest pain) are interpreted catastrophically, re-activating the vicious, self-perpetuating catastrophic cycle. Indeed, higher levels of pain catastrophizing have been associated with pain-related interpretation bias (Khatibi et al., 2014), while attentional and interpretation biases for pain-related stimuli have been observed in individuals with chronic pain (Schoth & Liossi, 2016; Schoth et al., 2019; Todd et al., 2015). These biases may play a major role in the onset and maintenance of chronic pain. Pain catastrophizing has been associated with reduced quality of life in heterogeneous groups of patients with chronic pain (Craner et al., 2016), in patients with severe hip osteoarthritis (Hayashi et al., 2019), chronic intractable pain of the trunk/ limbs mainly due to failed back surgery syndrome or radiculopathies (Rosenberg et al., 2015), chronic pelvic pain (Sewell et al., 2018), as well as in cancer survivors living with chronic pain (Poulin et al., 2016a, 2016b).

Another psychological factor that is related to emotion regulation and has been suggested to predict the intensity of both acute (Ruiz-Aranda, Salguero, & Fernandez- Berrocal, 2011) and chronic pain (Doherty et al., 2017) is emotional intelligence. Conceptually, EI is typically thought of as a multi-component construct that reflects the extent to which a person attends to, processes, and acts on the information of an emotional nature, intrapersonally and interpersonally (Kafetsios & Zampetakis, 2008; Mayer & Salovey, 1997). According to the four-branch model of emotional intelligence (Mayer & Salovey, 1997; Salovey & Grewal, 2005), this concept comprises the abilities to (a) perceive, decipher, monitor, and identify one’s own and others’ emotions accurately, (b) understand, discriminate, and recognize accurately different emotions, their outcomes, and trends over time, (c) use and deploy emotion-relevant information to facilitate thought, problem-solving, and decision making, and (d) regulate and manage emotions, in order to promote emotional and intellectual growth and achieve intended goals. Individuals with adequate affective information processing would be able to manage the negative affect (i.e., distress and unpleasurable engagement with the environment) generated by pain and use appropriate pain coping strategies and social support resources, hence reducing the perceived intensity of pain. High levels of emotional intelligence have been associated with better quality of life in patients with cancer (Baudry et al., 2019; Chen, Wang, Peng, & Zhu, 2021; Mirzaei et al., 2019; Rey et al., 2013). A large body of literature has documented the relationship between EI and better physical health through greater use of proactive self-care health practices, improved adherence to medical advice, better interactions with health care professionals, more frequent task-oriented coping to deal with health problems, greater social support, better stress management, and more positive emotions (e.g., Martins et al., 2010; Mikolajczak et al., 2015; Zeidner et al., 2012). Moreover, EI is related to lower pain intensity and reduced pain catastrophizing (magnification) in patients with chronic pain (Doherty et al., 2017), while EI has been associated with lower pain scores in patients with fibromyalgia (Luque-Reca et al., 2021).

Emotional intelligence has rarely been examined in relation to chronic pain and pain catastrophizing in patients with cancer. In addition, although PC and pain intensity have been assessed as explanatory variables related to patients’ quality of life, they have yet to be examined as mediators in the relationship between EI and QoL. Given the paucity of supporting evidence regarding the mediating role of PC and pain intensity in the relationship between EI and QoL, and following existing literature and the theoretical framework described by the FAM, the present study sought to investigate direct and indirect paths linking emotional intelligence, pain catastrophizing, chronic pain, and quality of life in cancer patients. We hypothesized that (a) quality of life would positively be related to emotional intelligence, negatively associated with pain catastrophizing, and negatively linked to the intensity of pain, (b) compared to patients with low pain catastrophizing, patients with high pain catastrophizing would have lower mean quality of life scores, (c) quality of life would significantly be associated with pain catastrophizing and pain intensity, after controlling for the effects of demographic and clinical variables, (d) pain catastrophizing and pain intensity would mediate the relationship between emotional intelligence and quality of life.

The secondary aim of the current study was to examine the factor structure of a multidimensional PC scale, given that the PC scale that was administered to study participants had not been validated in the Greek version in a sample of cancer patients with chronic pain through a hypothesis- or theory-driven method, such as confirmatory factor analysis (CFA). A better understanding of the pain experience in cancer survivors could help inform future health care educational priorities and psychological interventions to improve quality of life (e.g., Garland et al., 2019; Koh et al., 2018; Ngamkham et al., 2019).

## Methods

### Participants and Procedure

One hundred and five outpatients with cancer consecutively referred to and attending the pain clinics of three public hospitals and two palliative care units located in the capital city of Athens, Greece, were invited to participate in this cross-sectional study. To be selected, all patients had to meet the following criteria: (1) be fluent in the Greek language, (2) be adults under the age of 85 years, (3) have a current cancer diagnosis, and (4) have been diagnosed with pain that persisted for more than 3 months. Patients were excluded if they were not ambulatory, had severely impaired mobility (requiring the use of a walker), or were diagnosed with severe cognitive impairment. Participants were assured that they were free to participate or to decline to participate or to withdraw from the research at any time without giving reasons and without detriment to their care. Precautions were taken to secure anonymity, while informed consent was obtained from participants in the study after the nature of the research had been explained to them. The response rate was 84.7%, and the final sample consisted of 89 cancer patients with chronic pain who provided complete data on the measures included in the study. The institutional review boards approved the protocol at each registering institution.

### Measures

Demographic information included age, gender, level of education, marital status, and living arrangements. Medical information relating to time since cancer diagnosis and type of therapy for cancer pain was obtained from the patients’ medical records. The following measures were used to assess psychological variables.

#### Pain Intensity

Pain was assessed using the Short Form of the McGill pain questionnaire (SF-MPQ; Melzack, 1987). The SF-MPQ consists of 15 items, including 11 sensory and 4 affective descriptors, capturing the qualitative aspects of pain. All items are self-rated by the patient according to the pain intensity level, on a 4-point scale (0 = *none*, 1 = *mild*, 2 = *moderate*, 3 = *severe*). The higher the total score on the SF-MPQ, the more the patient's pain experience was negative, and the intensity of pain increased. In addition, the questionnaire includes (a) a visual analog scale (VAS) to determine the average pain intensity and (b) the present pain intensity (PPI), a six-point numerical and verbal rating scale to describe the overall present pain (Katz & Melzack, 2011). Only the total score obtained from the 15 items was used in the present study, while its internal consistency reliability was satisfactory (*α* = 0.75). The theoretical background of the SF-MPQ is provided by the biopsychosocial model of pain since it combines both the physiological and psychological dimensions of the pain experience (Gatchel et al., 2007; Katz & Melzack, 2011). The translation of the questionnaire into the Greek language and its validation were conducted by Georgoudis et al. (2000). They found satisfactory internal consistency reliability of the total scale (*α* = 0.71).

#### Quality of Life

The health-related quality of life of cancer patients was assessed using the QLQ–C30 (Version 3.0) of the European Organization for Research and Treatment of Cancer (EORTC; Aaronson et al., 1993; Fayers et al., 2001). The QLQ-C30 consists of 30 questions that cluster into subscales—both multidimensional and unidimensional—including 5 functional scales (*CF* Cognitive Functioning, *EF* Emotional Functioning, *PF* Physical Functioning, *RF* Role Functioning, *SF* Social Functioning), 3 symptom scales (*FA* Fatigue, *NV* Nausea/vomiting,* PA* Pain,), a global health status/ QoL scale, and 6 single symptom items (*DY* Dyspnea, *SL* Sleep Disturbances,* ΑΡ* Appetite Loss, *CO* Constipation, *DI* Diarrhea, *FI* Financial Impact). Most of the questions are answered on a 4-point Likert scale (1 = “*not at all,*” 2 = “*a little,*” 3 = “*quite a bit*,” 4 = “*very much*”), apart from the two items of the global health status scale, that are rated on a 7-point Likert scale that ranges from 1 = “*very poor*” to 7 = “*excellent.”* Respondents are asked to rate their functionality and symptoms during the past week. All scales and single-item measures are transformed to obtain scores from 0 to 100. A high scale score for the functional scale represents a high/healthy functioning level. A high score for the global health status represents high QoL, while a high score for a symptom scale or item represents a high symptomatology level. A total (summary) score can be computed from the mean of 13 of the 15 QLQ-C30 scales, where the symptom scales are reverse scored, while the global quality of life and the FI scales are not included in the total score. Higher total scores indicate better quality of life. The QLQ-C30 is a widely used psychometric tool within the interdisciplinary field of clinical trials; therefore, its utilization facilitates the comparison of findings across studies. Mystakidou et al. (2001) reported satisfactory validity and reliability of the Greek version of the QLQ-C30 in a sample of cancer patients receiving palliative care, with internal consistency reliability coefficients ranging from 0.71 to 0.79 for the functional scales.

#### Pain Catastrophizing

Pain catastrophizing was measured with the Pain Catastrophizing Scale (PCS; Sullivan, 2009; Sullivan et al., 1995). PCS is a 13-item instrument that asks participants to reflect on past painful experiences and indicate the degree to which they have each of a series of thoughts or feelings when in pain. All answers are rated on a 5-point scale, ranging from 0 = “*not at all*” to 4 = “*all the time*.” The PCS yields four scores: a total score of catastrophizing and three subscale scores, assessing the rumination (e.g., “I keep thinking about how much it hurts”), magnification (e.g., “I become afraid that the pain will get worse”), and helplessness (e.g., “It’s awful and I feel that it overwhelms me”) dimensions separately. Higher scores denote a higher degree of catastrophizing. The PCS as a full scale has been shown to be a reliable measure (Ikemoto et al., 2020; Wheeler et al., 2019). Various methods of differentiating between high and low levels of pain catastrophizing have been proposed and used, including the median split (Boonstra et al., 2016; Hadlandsmyth et al., 2019), the upper tertile approach (Birch et al., 2017; Riddle et al., 2010), and other splits at selected scale points (Dave et al., 2017; van Wyngaarden et al., 2021). However, a large number of investigators have adopted the cut-off value of 30 (Amtmann et al., 2020; Barjandi et al., 2021; Berube et al., 2019; Honkanen et al., 2021; Poulin et al., 2016a, 2016b; Sabo & Roy, 2019), initially suggested by Sullivan (2009), the creator of the PC scale. According to Sullivan (2009), a total PCS score of 30 represents clinically relevant level of catastrophizing, and this cut-off score corresponds to the 75th percentile of the distribution of the PCS scores in clinical samples of chronic pain patients. The same author argued that individuals who scored above the 75th percentile would be considered at high risk for the development of disability and chronicity (i.e., the pain condition would persist over an extended period of time) and would be suitable candidates for targeted intervention programs. Thus, we followed Sullivan’s suggestion and applied the cut point of 30. Regarding the Greek version of the PCS, although a previous PCS version was available in the Greek language (Papaioannou et al., 2009), data analysis had been based on exploratory factor analysis. Thus, a CFA was still lacking, and thus we explored the fit of one-, two-, and three-factor structures to the data. Formal permission for the translation and validation of the new Greek version was obtained from the MAPI Research Trust. PCS was translated from English into Greek, using the forward–backward technique, followed by pilot-testing and cognitive debriefing in order to ensure the clarity and comprehensibility of the items, to identify difficult, confusing, or upsetting/ offensive items, to test translation alternatives, to identify translation modifications, and to highlight inappropriate items or response options. In this way, conceptual equivalence and cultural relevance and adaptation were achieved.

#### Emotional Intelligence

Emotional Intelligence was measured using the Wong-Law Emotional Intelligence Scale (WLEIS; Wong & Law, 2002). This 16-item self-report questionnaire consists of four dimensions, each with four items, consistent with the four-branch model of interrelated skills proposed by Mayer and Salovey (1997). The Self-Emotion Appraisal dimension (SEA) assesses individuals’ self-perceived ability to understand their deep emotions and express these emotions naturally (e.g., “I have a good understanding of my own emotions”). The Others’ Emotion Appraisal dimension (OEA) relates to a person’s ability to perceive, monitor, and understand the emotions of other people in the social environment (e.g., “I am a good observer of others’ emotions”). The Use of Emotion (UOE) dimension refers to the self-perceived tendency of individuals to make use of their emotions by directing them toward constructive activities and performance (e.g., “I would always encourage myself to try my best”). Lastly, the Regulation of Emotion (ROE) aspect concerns individuals’ ability to regulate and control their emotions to enable more rapid recovery from psychological distress (e.g., “I am quite capable of controlling my own emotions”). The questionnaire items are rated on a 7-point Likert scale that ranges from 1 = “*strongly disagree*” to 7 = “*strongly agree.*” The WLEIS yields five scores: four subscale scores for each of the four dimensions of emotional intelligence mentioned above and one total EI score derived from the sum of the individual dimensions. Higher scores indicate a greater level of EI. Translation of the scale into Greek and validation of the Greek version of the WLEIS were conducted by Kafetsios and Zampetakis (2008) and Psilopanagioti et al. (2012). In the present study, internal consistency reliability was satisfactory for the full scale (*α* = 0.90), and the four subscales, namely SEA (*α* = 0.87), OEA (*α* = 0.70), UOE (*α* = 0.88), and ROE (*α* = 0.92).

### Data Analyses

Descriptive statistics were computed for demographic variables and pain intensity, QoL, pain catastrophizing, and emotional intelligence scales. Pearson correlation coefficients were used to explore linear relationships between variables. Independent-samples t tests were performed to compare mean values in QoL subscales for two groups of participants (with high PC vs. low PC). Levene’s test was used to examine whether the variances of QoL scales for the two groups could be assumed to be equal.

A confirmatory factor analysis involving PCS scores using robust maximum likelihood estimation (MLR) was conducted with the *lavaan* package in R (Rosseel, 2021). Based on previous research (Akbari et al., 2021; Chibnall & Tait, 2005; Kemani et al., 2019; Sullivan et al., 1995), three models were compared and tested in order to identify the best factor structure of PCS and evaluate model fit to the data: (a) a one-factor model in which all 13 PCS items were assumed to load on a single latent factor (i.e., pain catastrophizing), (b) an oblique two-factor solution in which the 9 magnification and helplessness items combined were hypothesized to load on one latent factor (named powerlessness), while the remaining 4 items loaded on the rumination factor, (c) a three-factor model, in which separate item sets representing rumination (4 items), magnification (3 items), and helplessness (6 items) were allowed to correlate. The following indices were used to assess goodness of fit of the models (Hu & Bentler, 1999): χ^2^/*df,* the root-mean-square error of approximation (RMSEA) accompanied by its associated 90% confidence interval (CI), the standardized root-mean-square residual (SRMR), the comparative fit index (CFI), and the Tucker–Lewis Index (TLI). Model fit was considered adequate when *χ*^2^/*df* ≤ 2*,* CFI, and TLI were greater than 0.95, SRMR was lower than 0.08, and RMSEA was lower than 0.06. Improvements to model fit were indicated by a decrease in the model Akaike information criterion (AIC) and the sample-size adjusted Bayesian information criterion (SABIC). Model modification indices (MI), being measures of predicted decrease in *χ*^2^ values if a single parameter was freed and the model was reestimated, guided the decisions to change the models and improve the fit. Respecification of model parameters involved freeing some aspects of the matrix of factor loadings and the covariance matrix of measurement errors in the observed variables initially specified to be fixed. Since modification indices are sensitive to sample size, the expected parameter change (EPC) value for each modification index, as a direct estimate of the size of misspecification for fixed parameters, was also examined in order to estimate how much the parameter was expected to change when it was to become free. Improvements to model fit were indicated by a significant decrease in *χ*^2^ values, an increase in CFI and TLI, and a decrease in the model AIC and SABIC.

Multiple linear regression analysis was applied to examine QoL as the outcome variable and pain intensity and PC as predictor variables, while potential covariates (age, gender, education, marital status, and time since cancer diagnosis) were included in the model. Gender was coded 1 for female and 0 for male. Marital status was coded as 1 for “married” and 0 for “not married” (i.e., single, widowed, divorced). Possible violations of the assumptions of linearity, normality, and homoscedasticity of residuals were examined. In order to identify influential cases and outliers, Mahalanobis’ and Cook’s distances and difference in fit values (DfFits) were computed and inspected, together with appropriate plots between standardized residuals and predicted values. Multicollinearity was tested by checking the variance inflation factor (VIF) and tolerance. Independence of error terms and sequential correlation of adjacent errors was tested through the Durbin–Watson statistic. Regression analysis was performed using SPSS v.23.

To examine the potential mediating role of PC and pain intensity in the relationship between EI and QoL, we used the PROCESS macro extension program for SPSS (Hayes, 2018). A heteroscedasticity consistent standard error and covariance matrix estimator (HC3) was used. In the case of mediation, bootstrapping has been recommended (MacKinnon et al., 2004) because the sampling distribution of the mediated (indirect) effect may not be normal (Shrout & Bolger, 2002). Bootstrapping, a non-parametric method based on resampling with replacement, was conducted, generating 5000 bootstrap samples. The bootstrap confidence intervals for indirect, direct, and total effects were estimated using the 2.5th and 97.5th percentiles of the bootstrap distribution values as the lower and upper bounds of the interval. If the 95% CI did not contain 0, the indirect effect was considered significant at the 0.05 level. The direct, total, and indirect effects were tested separately, given that a significant indirect effect might be detected even when the direct or total effect was not statistically significant (Rucker et al., 2011).

## Results

### Descriptive Statistics

Table 1 provides a summary of the sample characteristics. The sample consisted of 38 male (42.7%) and 51 female (57.3%) participants who came to the medical pain centers for prescription renewal and palliative care services. Participants ranged in age from 19 to 82 years (*M* = 56.44, *SD* = 14.82). The majority of the respondents had secondary education (40.4%), were married (53.9%), and living with their own family (59.6%). Mean time since cancer diagnosis was 5.61 years (*SD* = 5.72, *Mdn* = 3.0). Regarding treatment for cancer-related pain, all participants were taking analgesic medications (opioid or non-opioid). Mean duration of visiting the palliative care unit was 6.8 months (*SD* = 11.1, *Mdn* = 2.0).

**Table 1:** Sociodemographic characteristics of the study sample

Variable	*n* (%)
Gender	
Male	38 (42.7)
Female	51 (57.3)
Age	
19–29	7 (7.9)
30–39	5 (5.6)
40–49	12 (13.5)
50–59	22 (24.7)
60–69	24 (27.0)
≥ 70	19 (21.3)
Education	
Elementary	20 (22.5)
Junior high school	7 (7.9)
High school (Lyceum)	36 (40.4)
University	22 (24.7)
Postgraduate	4 (4.5)
Marital status	
Married	48 (53.9)
Single	22 (24.7)
Divorced	10 (11.3)
Widowed	9 (10.1)
Living arrangements	
Living alone	17 (19.1)
With parental family	6 (6.7)
With their own family	53 (59.6)
With relatives	5 (5.6)
With non-relatives	8 (9.0)

### Factor Structure of PCS

Using CFA, we assessed the fit of three models: a one-factor model in which the 13 PCS items loaded on a single latent factor (Model 1), a solution with two correlated factors, named powerlessness and rumination (Model 2), and a model with three correlated factors, representing rumination, magnification, and helplessness (Model 3). Initially, using the adjusted quantile method based on Mahalanobis distance, we searched for multivariate outliers. We found that no such outliers could be identified. Shapiro–Wilk’s test for assessing item univariate normality and Mardia’s skewness and kurtosis test for assessing multivariate normality of the data set indicated some departure from normality. Subsequently, we used the robust maximum likelihood (MLR) estimation procedure with corrected standard errors and test statistics. MLR performs well with categorized, Likert-type response, and non-normally distributed data, yielding relatively unbiased parameter estimates and standard errors, especially under conditions of small sample sizes and greater data asymmetry (Bandalos, 2014).

In order to use Δ*χ*^2^ and ANOVA to compare model fit meaningfully, a requirement is that models are nested (i.e., the parameters estimated in one model are a subset of the parameters in another model, for example, by fixing or constraining some free parameters or by imposing restrictions on a more general model, yielding a nested model; Bentler & Satorra, 2010). Although the values of the chi-square statistics obtained with standard ML estimator from nonhierarchical models can still be compared, the differences between them cannot be tested for significance. Akaike’s Information Criterion (AIC) allows such comparisons. We thus assessed whether the three models were nested, using the *nonnest2* package in R (Merkle, You, Schneider, & Bae, 2020). The variance tests indicated that the models were distinguishable from one another and non-nested. Moreover, based on the non-nested likelihood ratio tests (Merkle et al., 2016), we compared the fits of the three distinguishable models and found that the models had not equal fit in the population: M3 fitted better than M1 and M2. The AIC statistic was significantly lower for M3 than for M2 or M1, given that the AIC difference between M3 and M2 had 95% CI [− 44.75, − 1.65] that did not overlap with 0, implying that fit of M3 could be preferred over that of M2. The same hold true for the AIC difference between M3 and M1 with 95% CI [− 127.16, − 53.45].

Model 3 consisted of 3 factors with 13 indicators totally. With 13 observed variables, the number of observed unique variances and covariances was 91 (= 13 × 14 /2). In a model without any constrained parameters, there would be 29 freely estimated parameters (when scaling was carried out by fixing the factor variances): 13-factor loadings, 13 residual variances, and 3-factor covariances. Thus, for this model, degrees of freedom were equal to 62 (*df* = 91–29). For such a model, we calculated the power to reject close fit (*H*_0_: RMSEA ≤ 0.05) when in the population there was no close fit (*H*_1_: RMSEA = 0.10; Jak et al., 2021). We should note that Browne and Cudeck (1992, p.239) have suggested that RMSEA ≥ 0.10 indicates poor and unacceptable model fit. Then, the power to reject RMSEA of 0.05 (close fit of the model) was equal to 0.83. That is, the power to reject close fit when, in reality, there was not-close fit, with a sample size of 89, 62 *df*, and α = 5%, was satisfactory and equal to 0.83. Based on Daniel Soper’s calculator (Soper, 2021), and based on the number of observed (= 13) and latent variables (= 3) in Model 3, as well as on the ratio of the number of indicators to latent variables (= 4.33), the minimum sample size required to detect a large effect size of 0.5 for factor correlations, given the structural complexity of the model (Westland, 2010), was *N* = 89, to achieve statistical power = 0.80, at *α* = 0.05. Additionally, we used the PwR program (Wang & Rhemtulla, 2021) to estimate the power to detect each parameter of Model 3 via simulations. Based on the standardized values of the parameters (e.g., factor loadings, residual variances), with a sample size of *N* = 89, *α* = 0.05 and 5000 simulated samples, the estimated power for the model parameters ranged from 0.83 to 1, except that for the residual variance of item 6, which was lower than 0.80.

Thus, the study of Model 3 was confirmed to be adequately powered. The overall pattern of results presented in Table 2 illustrated that the 3-factor solution (Model 3) corresponded to the model with a better fit to the data, as indicated by the smaller RMSEA, SRMR, AIC, and SABIC values, and the higher CFI and TLI values. In addition, the SRMR was good and lower than the recommended threshold of 0.08, and the *χ*^2^/*df* was acceptable (< 2). Nevertheless, the remaining fit indices for M3 were indicative of inadequate model fit, given that the value of RMSEA (= 0.103) was above the recommended 0.06 cut-off, while the CFI and TLI values did not pass the 0.95 threshold. Because Model 3 was determined to fit the data inadequately based on quantitative analysis, we proceeded to examine potential modifications that could further improve the fit of the three-factor model. We respecified a new model (M3R) in which covariations between the residuals of items 8, 11, and 1 were added. A cross-loading item was also included (item 9 also loaded on the magnification factor, with standardized loading equal to 0.100,* p* = 0.026). This revised model resulted in a substantial improvement in the goodness-of-fit statistics, with Δ*χ*^2^ (4) = 34.79, *p* < 0.001. Revised model’s test statistic and fit indices were as follows: *χ*^2^ (58) = 75.88, *p* = 0.058, TLI = 0.970, CFI = 0.978, RMSEA = 0.059 (90% CI [0, 0.091]), SRMR = 0.049, AIC = 2948.03, SABIC = 2926.01. The test of close fit for RMSEA was not statistically significant at the 5% level (*p* = 0.326), which meant that we could not reject the null hypothesis that the RMSEA was less than or equal to 0.05. Thus, the revised model provided a much better fit to the data than the previous one (M3), as indicated by an increase in CFI and TLI (> 0.95), a decrease in RMSEA, SRMR AIC, and SABIC, and a significant reduction in the value of the *χ*^2^ statistic. Consideration of the measurement error correlations suggested that the covariance of these items that was unaccounted for by their latent factors was likely due to a method effect stemming from content overlap (e.g., “anxiously want the pain to go away,” “keep thinking about how badly I want pain to stop,” “worry about whether pain will end”). Similar secondary loadings and covariation between error terms in PCS items have been noticed by other researchers in the field (Osman et al., 1997, 2000).

**Table 2:** Confirmatory factor analysis of PCS items, and goodness-of-fit indices for three models

	*χ*^2^ (*df*)	*p*	CFI	TLI	RMSEA	SRMR	AIC	SABIC
Model 1 (M1)	204.08 (65)	< 0.001	0.841	0.810	0.162	0.077	3074.61	3057.26
Model 2 (M2)	139.49 (64)	< 0.001	0.913	0.894	0.121	0.068	3007.51	2989.49
Model 3 (M3)	115.09 (62)	< 0.001	0.939	0.923	0.103	0.057	2984.31	2964.96

*PCS* Pain Catastrophizing Scale, *df* degrees of freedom, *CFI* comparative fit index, *TLI* Tucker–Lewis index, *RMSEA* root-mean-square error of approximation, *SRMR* standardized root-mean-square residual, *AIC* Akaike information criterion, *SABIC* sample-size adjusted Bayesian information criterion

**Fig. 1:**
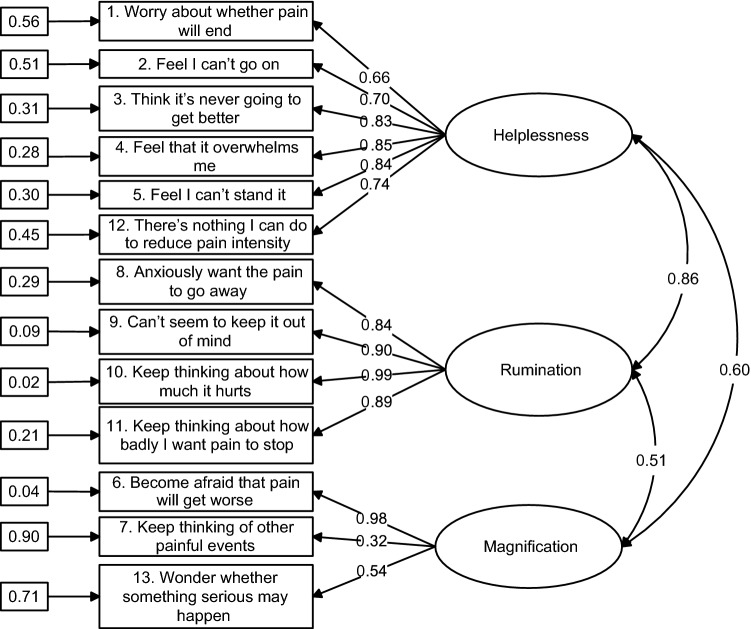
Standardized solution for the revised Model 3 (model M3R) with three correlated first-order factors based on confirmatory factor analysis. Numbers enclosed in rectangles indicate measurement errors and those in the middle of straight lines indicate factor loadings. Curved lines indicate correlations

Figure 1 shows the completely standardized solution for the revised 3-factor measurement model. In this diagram, observed variables are represented by rectangles, and latent variables are enclosed in ellipses. Standardized factor loadings ranged from 0.54 to 0.99, except that for item 7, whereas 77% of them were large (≥ 0.70). Most of the standard errors of the unstandardized parameter estimates were small (≤ 0.10), indicating that the values of the model parameters had been estimated accurately. All unstandardized factor loadings were statistically significant at the 0.1% level. All measurement errors in the observed variables were statistically significant at the 1% level, except the error variance for items 6 and 10 (with unstandardized estimates equal to 0.07 and 0.04, respectively, *p* > 0.05). Inspection of the squared multiple correlations for the observed variables indicated that approximately 69% of them were relatively high (≥ 0.50), meaning that less than half of their variance was unique and thus unexplained by the factor each variable was specified to measure. Accordingly, the majority of the observed variables were good measures of their latent variables. The three latent factors were positively and significantly correlated to each other, with correlations ranging from 0.51 to 0.86, *p* < 0.001. Reliability indices (omega; Raykov, 2001) of the three factors corresponding to rumination, magnification, and helplessness were estimated to be equal to 0.944, 0.709, and 0.890, respectively. These results indicated that the pain catastrophizing scale could be represented by three reliable and correlated latent dimensions, taking error term covariation into account.

### Correlations Among Study Variables

Table 3 presents correlation coefficients between the main psychological variables included in the analyses. Better patients’ QoL was significantly correlated with higher EI (*r* = 0.506, *p* < 0.001), lower pain catastrophizing scores (*r* = − 0.607, *p* < 0.001), and lower pain intensity (*r* = − 0.724, *p* < 0.001). Among the remaining correlations, there was a noteworthy finding: although three of the four dimensions of EI were negatively and significantly related to pain intensity, the OEA dimension was not significantly correlated with pain intensity (*r* = − 0.105, *p* > 0.05), meaning that patients’ appraisal and understanding of emotions of others were not significantly associated with their own experience of pain intensity.

**Table 3:** Mean values, standard deviations, and correlation coefficients between psychological variables

	*M*	*SD*	1	2	3	4	5	6	7	8
1. Quality of Life (total)	57.66	17.40	1							
2. Pain Catastrophizing (total)	23.58	12.49	− 0.607**	1						
3. Pain Intensity (total)	20.00	8.74	− 0.724**	0.650**	1					
4. Emotional Intelligence (total)	73.61	17.07	0.506**	− 0.559**	− 0.423**	1				
5. SEA	19.91	5.38	0.322**	− 0.383**	− 0.294**	0.805**	1			
6. ROE	16.11	6.33	0.425**	− 0.504**	− 0.386**	0.803**	0.524**	1		
7. UOE	16.88	6.45	0.501**	− 0.496**	− 0.436**	0.827**	0.478**	0.571**	1	
8. OEA	20.82	3.83	0.235*	− 0.272*	− 0.105	0.609**	0.540**	0.195	0.397**	1

*SEA* Self-Emotion Appraisal, *ROE* Regulation of Emotion, *UOE* Use of Emotion, *OEA* Others’ Emotion Appraisal

*p < 0.05; **p < 0.01

### Comparisons Between PC Groups

Table 4 presents comparisons between two groups of participants (with high PC vs. low PC) in mean QoL scores. Patients with high PC levels scored significantly lower on the five functional QoL scales (i.e., PF, RF, CF, EF, SF) and on global health status and the total (summary) QoL score than patients with low PC levels. Moreover, those with high PC levels scored significantly higher on three symptom scales/ items, namely pain, fatigue, and insomnia.

**Table 4:** Comparisons between participants with high PC vs. low PC in mean QoL values

QoL scales	Pain catastrophizing		
	Low PC (*n* = 60)	High PC (*n* = 29)	*t*-test
Physical functioning	56.67 (24.69)	29.65 (19.56)	5.16***
Role functioning	41.39 (33.12)	12.07 (15.36)	5.70***
Social functioning	53.89 (32.67)	13.79 (21.39)	6.92***
Emotional functioning	59.58 (24.35)	37.64 (23.32)	4.04***
Cognitive functioning	82.78 (18.66)	53.45 (27.23)	5.24***
Fatigue	53.89 (26.50)	78.16 (24.93)	− 4.13***
Nausea/ vomiting	10.00 (19.45)	18.96 (22.15)	− 1.95
Pain	61.67 (26.27)	85.63 (16.50)	− 5.24***
Dyspnea	36.11 (33.21)	41.38 (38.48)	− 0.67
Insomnia	31.11 (35.71)	55.17 (35.94)	− 2.97**
Appetite loss	26.67 (33.50)	40.23 (36.05)	− 1.75
Constipation	32.22 (37.31)	43.68 (43.74)	− 1.28
Diarrhea	10.00 (22.38)	5.75 (12.81)	1.14
Financial difficulties	41.67 (32.26)	48.27 (34.02)	− 0.89
Global health status	53.89 (21.17)	30.46 (23.70)	4.70***
Total (summary) score	64.05 (15.32)	44.43 (13.70)	5.85***

*QoL* Quality of Life; *PC* Pain Catastrophizing

**p < 0.01; ***p < 0.001

### Regression Analysis

To build the regression model with QoL as the outcome variable, forced entry was used to develop the regression equation. Table 5 shows that the only variables that appeared to have unstandardized regression coefficients significantly different from zero in predicting QoL were education, pain intensity, and pain catastrophizing. The positive coefficient for the education variable (*B* = 2.915, *p* = 0.017) meant that patients who had higher education experienced higher QoL, controlling for the effects of the other predictor variables. PC related significantly and negatively to QoL (*B* = − 0.391, *p* = 0.004), suggesting that the higher the pain catastrophizing score, the worse the QoL. Moreover, the negative sign of the regression coefficient for pain intensity (*B* = − 1.133, *p* < 0.001) meant that survey respondents who had higher levels of pain intensity had poorer QoL. This variable had the greatest standardized coefficient (in absolute value) and appeared to be the best predictor of QoL, at least among the variables included in the equation. The model *R*^2^, when only the demographic and clinical variables were in the model, was equal to 0.128 (adjusted *R*^2^ = 0.075). There was a statistically significant improvement (*R*^2^ change = 0.478) in the relationship between the set of independent variables and the dependent variable, when the psychological variables (pain intensity and PC) were included, *F* change (2, 81) = 49.202, *p* < 0.001. The proportion of variance in the dependent variable (QoL) explained by the seven independent variables was 60.6% (adjusted *R*^2^ = 0.572), *F*(7,81) = 17.803, *p* < 0.001. The Durbin–Watson statistic was equal to 2.065, a value very close to 2 and beyond the upper bound of the interval [1.49, 1.83] established for the critical values corresponding to our sample size and the number of predictors, indicating that there was no autocorrelation present in the error, at the 5% level (Chatterjee & Hadi, 2012). Multicollinearity was not detected, as all independent variables had large tolerance values (> 0.50) and small VIF values (< 2). Using the G*Power program (Faul, Erdfelder, Buchner, & Lang, 2009), post hoc statistical power analysis indicated that the achieved power of the test for the increase in *R*^2^ (= 0.478) due to the addition of the two psychological variables (pain intensity, PC) in the model, given a 5% level and a noncentrality parameter *λ* = 59.674, was very high (= 0.999).

**Table 5:** Final model regarding regression analysis with QoL as the dependent variable

Variables	*B*	*SE*	β	*t*	*p*	95% CI	
						*LL*	*UL*
Age	0.013	0.101	0.011	0.129	0.898	− 0.188	0.214
Gender	4.003	2.728	0.114	1.467	0.146	− 1.425	9.431
Education	2.915	1.200	0.201	2.429	0.017	0.527	5.302
Marital status	2.063	2.559	0.059	0.806	0.422	− 3.027	7.154
Time since cancer diagnosis	− 0.033	0.223	− 0.011	− 0.149	0.882	− 0.477	0.410
Pain intensity	− 1.133	0.194	− 0.569	− 5.845	< 0.001	− 1.519	− 0.747
Pain catastrophizing	− 0.391	0.132	− 0.281	− 2.953	0.004	− 0.655	− 0.128

*CI* confidence interval, *LL* lower limit, *UL* upper limit

### Mediation Analysis

**Fig. 2:**
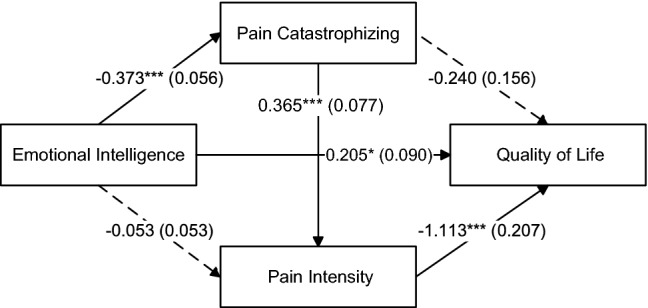
Mediation model being tested. Unstandardized parameter estimates and standard errors (in parentheses) for direct effects among psychological variables. Dashed arrows indicate nonsignificant paths.*p < 0.05; ***p < 0.001

Figure 2 displays the serial multiple mediation analysis results, with EI as the independent variable and QoL as the outcome variable, taking the contribution of covariates (age, gender, education) into account. The first analysis concerning the mediating role of PCS in the relationship between EI and QoL revealed that EI was negatively related to PC (*B* = − 0.373, *t* = − 6.611, *p* < 0.001), which in turn was negatively but not statistically significantly associated with QoL (*B* = − 0.240,* t* = − 1.545, *p* = 0.126). The direct effect of EI on QoL was positive and statistically significant (*B* = 0.205, *t* = 2.269, *p* = 0.026)*,* with a completely standardized coefficient equal to 0.201. The indirect effect of EI on QoL through PC was not statistically significant, with a completely standardized coefficient equal to 0.088 (*SE* = 0.057) and 95% CI [− 0.019, 0.205].

Regarding the second analysis involving the indirect effect of pain intensity in the relationship between EI and QoL, EI was negatively but not statistically significantly related to pain intensity (*B* = − 0.053, *t* = − 1.010, *p* = 0.315), which in turn was negatively associated with QoL (*B* = − 1.113, *t* = − 5.381, *p* < 0.001). The indirect effect of EI on QoL exerted through pain intensity was not statistically significant, with a completely standardized coefficient equal to 0.058 (*SE* = 0.057) and 95% CI [− 0.050, 0.180], which led to the failure to reject the null hypothesis that the indirect effect was zero. At this point, we should note that EI also had significant and negative indirect effects on pain intensity through PC, with a completely standardized coefficient equal to − 0.265 (*SE* = 0.060) and 95% CI [− 0.394, − 0.159], signifying that PC served as a significant full mediator of the EI–pain intensity relationship. We should also emphasize that, among the three dimensions of the PCS, only helplessness and rumination had significant indirect effects. The path from magnification to pain intensity was not statistically significant (*B* = 0.392, *t* = 1.216, *p* = 0.228). The indirect effect of magnification in the relationship between EI and QoL was also not statistically significant, with a completely standardized coefficient equal to 0.012 (*SE* = 0.033) and 95% CI [− 0.052, 0.084] that contained zero.

In the final analysis, we assumed that the relationship between EI and QoL was mediated simultaneously by both PC and pain intensity. PC was positively related to pain intensity (*B* = 0.365, *t* = 4.733, *p* < 0.001), with a completely standardized coefficient equal to 0.521. Although the direct effect of PC on QoL was not statistically significant, there was a statistically significant indirect effect through pain intensity, with a negative completely standardized coefficient equal to -0.291 (*SE* = 0.075) and 95% CI [− 0.454, − 0.162]. The specific indirect effect of EI on QoL through PC and pain intensity was positive and statistically significant, with a completely standardized coefficient equal to 0.148 (*SE* = 0.043) and 95% CI [0.074, 0.248]. This interval did not contain zero, suggesting that the indirect effect was statistically significant at the 5% level. Thus, PC and pain intensity jointly emerged as significant mediators of the EI–QoL relationship. EI was significantly related to lower PC scores, which in turn were associated with better QoL through decreased pain intensity scores. The total indirect effect of EI on QoL through PC and pain intensity was positive and statistically significant, with a completely standardized coefficient equal to 0.294, 95% CI [0.164, 0.440]. The total (direct and indirect) effect of EI on QoL was positive and statistically significant (*B* = 0.505, *SE* = 0.091, *t* = 5.551, *p* < 0.001), with a completely standardized coefficient equal to 0.495. We should note that among the separate dimensions of PC, only rumination and helplessness (but not magnification) were found as significant multiple mediators of the EI–QoL relationship. The completely standardized coefficient for magnification as a multiple mediator was equal to 0.030 (*SE* = 0.027) and 95% CI [− 0.017, 0.089], a confidence interval that straddled zero, suggesting insufficient evidence that EI affected QoL through magnification.

## Discussion

This cross-sectional study mainly examined direct and indirect relationships between emotional intelligence, pain catastrophizing, pain intensity, and quality of life in cancer patients with chronic pain. A secondary objective of this study was to investigate the factor structure of the PCS using CFA. Results provided support for our research hypotheses. More specifically, our first hypothesis, that higher QoL scores would be related to higher EI scores and lower PC and pain intensity scores, was confirmed. This finding aligns with previous research (Baudry et al., 2019; Hayashi et al., 2019; Mirzaei et al., 2019). Our second hypothesis, namely that patients with high PC would have poorer QoL, was supported. Relative to patients with low PC, those with high PC (i.e., with a strong tendency to describe the pain experience in more exaggerated terms than the average person, to ruminate on it more, and to feel more helpless about the experience) had significantly lower scores in all functional QoL scales (i.e., PF, RF, EF, CF, SF), global health status, total (summary) score, and three symptom items or scales (namely, pain, fatigue, and insomnia). This finding is congruent with that of Dasiran and Akbas (2021), who found that higher levels of PC were associated with worsening QoL (physical and mental dimensions) in patients who had been surgically treated for breast cancer. According to the FAM, pain catastrophizing can lead to fear about activity and to avoidance behavior, leading to functional disability and mood disturbance, negatively impacting QoL.

The present results also lend support to our third hypothesis, showing that poorer QoL was significantly associated with increased PC scores and heightened pain intensity, after controlling for the effects of demographic and clinical variables. This finding adds to the existing empirical evidence that PC and pain intensity are independently associated with poorer QoL (Goncalves et al., 2021; Hayashi et al., 2019; Laroche et al., 2017; Shim et al., 2018). Moreover, we found that those who had higher education experienced higher QoL, a finding that is compatible with relevant literature (Gil-Lacruz et al., 2020). A higher level of education is related to better health indicators, a better understanding of health information, better cognitive skills associated with personal care, better access and use of health resources, better use of social networks, and ultimately to better QoL. Turning to our final hypotheses about the direct and indirect paths from EI to QoL and the mediating role of PC and pain intensity, it should be noted that EI had a positive and significant direct and indirect effect on QoL. This finding is consistent with study results by Hood et al. (2012) who found that pain catastrophizing mediated the relationship between positive traits and pain report. We argue that higher scores of EI are associated with lower PC scores, and these lower PC levels can lead to the experience of reduced pain intensity, which in turn is translated into better QoL. This may be a mechanism linking EI to enhanced QoL, while PC (helplessness and rumination dimensions) and pain intensity combined are important mediators, partially transmitting the effect of EI on QoL. These findings can be interpreted as follows.

In line with research findings by Ruiz-Aranda et al. (2011), emotional intelligence was not directly related to pain intensity. Its negative effect was mediated by PC. According to the previous work of Crombez et al. (1998) and the Cognitive–Affective Model of chronic pain (Eccleston & Crombez, 1999), catastrophic thinking about pain is linked to high somatic awareness and hypervigilance for threatening somatic symptoms and an inability to suppress or divert attentional focus away from pain-related thoughts, leading to rumination about pain, fear of more pain, withdrawal from social contacts, and higher levels of disability. Under these conditions, chronic pain constitutes a source of continuous distress and arousal that disrupt ongoing thought, attention, and behavior, and impose new action priorities. Following the attentional bias of pain catastrophizing and according to Fredrickson’s (2001, 2004) broaden-and-build theory of positive emotions, Ong (2010) has suggested that positive affectivity (associated with emotion regulation brought about by EI) may diminish stress exposure, ameliorate the adverse effects of stress, attenuate the cognitive narrowing engendered by pain catastrophizing, and strengthen the coping resources of individuals against pain and other stressors (Fredrickson & Joiner, 2002, 2018; Fredrickson et al., 2003). Positive emotions are believed to improve psychological resilience, put negative emotions in a broader context, increase coping resources, make it easier to see positivity in future situations, increase feelings of well-being, improve social integration, promote greater distress tolerance, and better emotion regulation. As a set of adaptive emotional abilities, emotional intelligence has been found to facilitate stress resilience (Schneider et al., 2013) and predict higher levels of positive emotions (Rey et al., 2013; Szczygieł & Mikolajczak, 2017). In this regard, emotional intelligence can decrease the frequency and severity of pain-related catastrophic thoughts by potentiating positive affectively and enhancing cognitive flexibility, leading to the experience of lower pain intensity and, ultimately, to better QoL. Decreased levels of pain catastrophizing (Lamé et al., 2005), and higher levels of resilience (Ristevska-Dimitrovska et al., 2015; Zhang, Zhao, Cao, & Ren, 2017) have been found to predict better QoL in patients with cancer. According to our findings, higher levels of emotional intelligence are related to lower levels of pain catastrophizing, leading to reduced pain intensity and better quality of life. Hence, our hypothesis that pain catastrophizing and pain intensity would mediate the effect of cancer patients' emotional intelligence on their QoL was confirmed. Thus, EI emerged not only as a protective factor against poor QoL, but it also might protect a person from high PC and the resulting elevated pain intensity. EI may be related to better social interactions and promote social functioning and perceived social support, which may positively influence mental health and overall QoL.

Moreover, pain catastrophizing was highly and positively related to chronic pain intensity. This finding is consistent with that of previous studies, suggesting a direct and significant effect of pain catastrophizing on the intensity of the reported pain (Parr et al., 2012; Poulin et al., 2016a, 2016b). According to the Fear-Avoidance Model of Pain, a high degree of pain catastrophizing enhances attenuation to the painful sensation, ultimately exacerbating its intensity (French et al., 2007; Leeuw et al., 2007). Finally, there was no significant direct path from PC to QoL, but there was a negative and significant indirect effect of PC on QoL through pain intensity, where pain intensity emerged as a significant mediator of the PC–QoL relationship.

Regarding the secondary aim of our study about the factor structure of the PCS, results showed that the three-factor model of the pain catastrophizing scale demonstrated the best fit to the data obtained from our sample of cancer patients experiencing chronic pain. All items of the three-factor model loaded on their original factors, supporting previous research that the PCS assesses three correlated yet different dimensions of pain catastrophizing (helplessness, magnification, and rumination) in patients with chronic pain (Sullivan, 2009). The present study results are alongside previous studies conducted by Cano et al. (2005), and van Damme et al. (2002) on samples of patients with low back injuries. However, the results are in contrast with that of Chibnall and Tait (2005), who found that a two-factor model of the PCS was more parsimonious than the three-factor or rival models. This inconsistency indicates that the questionnaire items may not have the same meaning for all groups of patients in different countries. Such discrepancies might be related to the different samples used in these studies (e.g., patients with fibromyalgia or chronic lower back pain). It is also possible that cultural differences can explain such discrepancies.

### Study Limitations

Τhe results of this study must be viewed in light of some limitations, which also suggest possible directions for further research. First, results must be considered within the context of our cross-sectional design. Although we have described the direct and indirect effects of the study variables, this causal language is a convention of the statistical approach, and relationships between variables do not imply directionality or causality between them. Thus, despite the importance of our research results indicating the role of emotional intelligence in reducing PC and improving QoL, longitudinal studies are needed to understand the sequence and causal nature of the relationships between variables. Second, another possible limitation stems from the use of self-report measures. Despite the high levels of reliability and validity of the scales, self-report measures are per se subjective and vulnerable to bias. Hence, multisource and multimethod strategies of collecting data could facilitate the objectivity of measures and limit method variance. Third, the practice of dichotomization of the continuous PC variable in order to test one of our research hypotheses is associated with some negative consequences, including loss of information about individual differences, loss of effect size and power, and risk of overlooking nonlinear effects (MacCallum et al., 2002). Taxometric analyses, using, for example, cluster analysis or latent class analysis, should be conducted to empirically support the existence of distinct groups of individuals with high or low PC within the observed sample, along with a clinically meaningful scale point that differentiated the groups. Fourth, replication of our study in a larger sample, gathering data on more clinical variables that may affect the pain experience and intensity (e.g., primary pain site and etiology, type of pain, type of pain management, duration of cancer-related pain, primary tumor site and stage), is warranted. In this regard, we should acknowledge that recent literature indicates that patients and the public report a number of significant concerns about the sharing of health data for research purposes, including breaches of confidentiality, security, and privacy, as well as about potential commercial misuse, access to and abuse of the data by third parties (e.g., insurance or pharmaceutical companies; Brall et al., 2021; Kalkman et al., 2022; Patil et al., 2016). Fear of discrimination, stigmatization, exploitation, or other repercussions as a consequence of data sharing have been widely reported by patients. Finally, according to the fear-avoidance model of chronic pain, catastrophic thinking deteriorates the painful sensation by causing avoidance behavior and enhancing hypervigilance. In the present study, we did not examine the relationship of emotional intelligence with the factors mentioned above, an area that we consider to be of interest for future investigation.

### Strengths and Implications

Despite the limitations, the present study suggests a novel conceptualization and mechanism for understanding the interaction between EI and QoL in cancer patients with chronic pain. Pain catastrophizing has been previously identified as a significant psychosocial variable in the pain experience. However, the present study expands on the current literature by demonstrating that emotional intelligence can both, directly and indirectly, be related to QoL via PC and pain intensity. These results may have important theoretical and clinical implications. Firstly, emotional intelligence may be a useful construct and means of identifying individuals who experience difficulties with the emotional management of pain. Thus, they would benefit from a psychological intervention to enhance their emotional intelligence (Hodzic et al., 2018; Kotsou et al., 2019) and influence the associated pain experience and QoL. However, we should note that certain dimensions of EI may show higher training effects and may have higher potential to be taught through interventions aimed at improving EI. For example, Hodzic et al. (2018) found that EI trainings were more effective for the “understanding emotions” dimension of EI (which includes awareness and knowledge about how emotions change over time, how they differ, how they combine together, how they link to situational factors, as well as understanding their outcomes, and identifying the causal relationships between events and emotions) than for the other dimensions (e.g., emotion perception, emotion facilitation of thought, or emotion regulation/ management). Gilar-Corbi, Pozo-Rico, Sanchez, and Castejon (2019) found that intrapersonal EI, self-perception, general mood, self-expression, and stress management were maintained after the completion of a training program on EI. On the other hand, improvements in emotional understanding and emotion management had strengthened over time. However, the results also revealed that training had a nonsignificant impact on interpersonal and adaptability skills. Furthermore, consideration of emotional abilities concerning catastrophic cognitions and quality of life may improve our understanding of individual differences in cancer pain intensity. Moreover, emotional intelligence appears to be a variable that can potentially be included in the fear-avoidance model of chronic pain as a factor that weakens the adverse effects of pain catastrophizing on pain intensity. The significant and negative association found between emotional intelligence and pain catastrophizing suggests that emotional competencies affect emotional information processing and the management of negative cognitions. As a result, a critical factor in reducing chronic cancer pain intensity through pain catastrophizing would be to empower the emotional abilities of patients in order to decrease the catastrophic orientation of thinking and its effect on pain (Bishop & Warr, 2003; Poulin et al., 2016a, 2016b) and quality of life (Lamé et al., 2005). Day et al. (2020) have found that changes in cognitive content and pain catastrophizing through enhanced perceived control of pain are significantly associated with pain interference improvement in patients with chronic low back pain involved in diverse pain psychosocial interventions (e.g., cognitive therapy, mindfulness meditation, mindfulness-based cognitive therapy). Thus, emotional intelligence could be encompassed into the theoretical and clinical protocol of psychological interventions as a factor that could reduce the frequency of catastrophic thinking and improve the quality of life of cancer patients with chronic pain. This possibility deserves further empirical evaluation in studies using experimental designs to elaborate on cancer pain assessment and management's existing knowledge.

Secondly, pain catastrophizing is responsive to psychosocial treatment interventions, such as cognitive-behavioral therapy (CBT; Ehde et al., 2014), emotional disclosure, graded activity and graded exposure, reassurance and activity encouragement (Wideman & Sullivan, 2011), mindful-based interventions (Simmons et al., 2019), multimodal treatment, and acceptance and commitment therapy (Schütze et al., 2018). Sullivan (2009) has suggested that individuals who obtain high scores on the PCS would be considered suitable candidates for a risk-factor-targeted intervention program. He has also emphasized that interventions aimed at minimizing catastrophic thinking will need to incorporate strategies for assisting catastrophizers in disengaging their attention from their pain symptoms. Indeed, prior research implicates the construct of pain catastrophizing as a therapeutic factor that contributes to the effectiveness of CBT-focused interventions. Furthermore, analyses examining relationships between pain catastrophizing and outcomes tend to bypass the unique influence of the different dimensions of pain catastrophizing and simply examine the construct as a whole. We claim that simply collapsing the three dimensions of this phenomenon (i.e., rumination, magnification, helplessness) may conceal nuanced relationships between specific dimensions of catastrophizing and outcomes that might inform treatment approaches. Results of our mediation analysis revealed that helplessness and the tendency to ruminate about pain mediated changes in all outcomes examined (i.e., pain intensity and QoL) beyond the effects of the other covariates tested in the models (i.e., age, gender, education). Interestingly, the mediating effect of magnification in the EI–QoL relationship was not statistically significant. Changes in the other two dimensions of pain catastrophizing—the tendency of an individual to feel helpless and perseverate in the face of pain—had the most significant impact on QoL. The tendency to ruminate over pain and feel helpless in response to it may be more debilitating than simply magnifying or exaggerating the impact of pain. Another possibility is that the tendency to magnify pain experience is more problematic earlier throughout pain chronicity and that reduction in the tendency to magnify and exaggerate the experience of pain becomes less impactful over time. Gilliam et al. (2017) examined the unique effects of the three subdomains of PC on changes in pain outcomes in chronic pain patients undergoing an interdisciplinary pain rehabilitation program. They found helplessness and rumination to be promising targets in improving treatment outcomes. Future research examining how these distinct constructs regarding PC may function as treatment mechanisms is a promising area of further study.

Thirdly, pain and pain intensity may be a target for “third- wave” cognitive and behavioral therapies, including mindfulness-based cognitive therapy, dialectical behavior therapy, and acceptance and commitment therapy (Georgescu, Dobrean, & Predescu, 2018; Dimidjian et al., 2016; Hadlandsmyth et al., 2019; Hayes & Hofmann, 2021; Roslyakova et al., 2021). More specifically, acceptance and commitment therapy shifts focus from pain reduction and control gain over one’s inner world to disability improvement and better life functioning through psychological flexibility, active acceptance of one’s worrisome thoughts, strong emotions, aversive memories, and pain sensations, thus removing the suffering aspect of the pain experience. Moreover, acceptance and commitment therapy promotes commitment to behavior change in accordance with one’s values (Karekla et al., 2018). In this regard, protocols for acceptance and commitment therapy should guide tailored interventions to treat chronic pain and consequently improve quality of life of cancer patients (Moreno et al., 2022; Smith et al., 2022).

## Conclusion

In summary, our study results suggest that patients with severe PC have significantly lower QoL functional scores and higher pain, fatigue, and insomnia symptoms, that EI and PC are independently related to poorer QoL, and that PC and pain intensity jointly act as significant mediators of the EI–QoL relationship. In addition, the PCS assesses three correlated yet different dimensions of pain catastrophizing (helplessness, magnification, and rumination) in cancer patients with chronic pain. These findings confirm the importance of accounting for psychological factors, such as EI and PC, when evaluating QoL in cancer patients with chronic pain, and may inform psychological interventions that can enhance EI, reduce pain catastrophizing, and minimize the negative impact (e.g., high pain intensity) of pain catastrophizing on QoL.

## Data Availability

The datasets used and/or analyzed during the current study are available from the corresponding author on reasonable request.

## Declarations

### Research Involving Human and Animals Rights

All procedures performed in studies involving human participants were in accordance with the ethical standards of the institutional research committee of the Palliative Care Units and with the 1964 Helsinki Declaration and its later amendments or comparable ethical standards. This article does not contain any studies with animals performed by any of the authors.
